# Identifying Two Common Types of Breast Benign Diseases Based on Multiphoton Microscopy

**DOI:** 10.1155/2018/3697063

**Published:** 2018-04-17

**Authors:** Yan Wu, Yuxiang Lin, Yuane Lian, Peihua Lin, Shu Wang, Fangmeng Fu, Chuan Wang, Jianxin Chen

**Affiliations:** ^1^Mathematics and Physics Institute, Fujian University of Technology, Key Laboratory of OptoElectronic Science and Technology for Medicine of Ministry of Education, Fujian Normal University, Fuzhou, Fujian 350118, China; ^2^Department of Breast Surgery, The Affiliated Union Hospital of Fujian Medical University, Fuzhou, Fujian 350001, China; ^3^Department of Pathology, The Affiliated Union Hospital of Fujian Medical University, Fuzhou, Fujian 350001, China; ^4^Key Laboratory of OptoElectronic Science and Technology for Medicine of Ministry of Education, Fujian Provincial Key Laboratory for Photonics Technology, Fujian Normal University, Fuzhou, Fujian 350007, China

## Abstract

Multiphoton microscopy has attracted increasing attention and investigations in the field of breast cancer, based on two-photon excited fluorescence (TPEF) and second-harmonic generation (SHG). However, the incidence of breast benign diseases is about 5 to 10 times higher than breast cancer; up to 30% of women suffer from breast benign diseases and require treatment at some time in their lives. Thus, in this study, MPM was applied to image fibroadenoma and fibrocystic lesion, which are two of the most common breast benign diseases. The results show that MPM has the capability to identify the microstructure of lobule and stroma in normal breast tissue, the interaction of compressed ducts with surrounding collagen fiber in fibroadenoma, and the architecture of cysts filled with cystic fluid in fibrocystic disease. These findings indicate that, with integration of MPM into currently accepted clinical imaging system, it has the potential to make a real-time diagnosis of breast benign diseases in vivo, as well as breast cancer.

## 1. Introduction

Multiphoton microscopy (MPM) has opened up the new possibilities for in vivo imaging biological tissues at the cellular and subcellular levels with high spatial resolution, by taking advantage of the natural fluorescing and optical properties of intrinsic biomolecules [1–3]. It displays several advantages over traditional imaging technique, such as being label-free, deep tissue imaging, and low photobleaching and phototoxicity, based on two-photon excited fluorescence (TPEF) and second-harmonic generation (SHG) [4–6]. Within breast tissue, many endogenous fluorophores including collagen bundles, nicotinamide adenine dinucleotide hydrogen (NAD(P)H), and flavin adenine dinucleotide (FAD) can easily generate strong TPEF signals, and collagen fibers with noncentrosymmetric molecular structure are more effective in producing SHG signals. Therefore, MPM attracts increasing attentions and investigations in the field of breast cancer including observing tumor initiation, monitoring tumor progression, and detecting tumor metastasis [7–10]. However, to the best of our knowledge, literatures focusing on application of MPM in breast benign disease are very limited.

As well as we know, breast benign diseases are the most common cause of breast problems; up to 30% of women suffer from breast benign diseases and require treatment at some time in their lives [11]. The incidence of breast benign diseases is about 5 to 10 times higher than breast cancer. Among breast benign diseases, fibroadenoma is the commonest that can occur in women of any age, with a peak incidence in the second and third decades of life [12]. Fibrocystic disease of the breast is the next common whose incidence increases with increasing age [11]. Thus, identifying optical diagnostic features of fibroadenoma and fibrocystic disease, as well as breast cancer, is absolutely essential for MPM to make real-time noninvasive diagnosing of breast diseases. In this study, MPM was applied to investigate the microstructure of fibroadenoma and fibrocystic disease. Moreover, several qualitative diagnostic parameters were extracted from the high-contrast TPEF/SHG images to demonstrate the obvious morphological alterations in these two breast benign diseases.

## 2. Materials and Methods

### 2.1. Sample Preparation

This study was approved by the Institutional Review Board of the Affiliated Union Hospital of Fujian Medical University (Fuzhou, China) and informed consent was obtained from each patient who participated. We followed the methods of Wu et al. 2015 [1]. A total of 12 samples (6 fibroadenomas and 6 fibrocystic diseases) from 12 different patients were collected after resection from patients. Normal tissues were 6 cm away from the diseased margin. Every specimen was cut into five serial slices with thickness of approximately 10 *μ*m by cryostat microtome. The middle slice was stained with hematoxylin and eosin (H&E) for histological images and the rest of the sections were sandwiched between the microscope slide and a cover slip for multiphoton microscopic imaging. To avoid dehydration or shrinkage during the imaging process, a small amount of phosphate-buffered saline solution was dripped onto the tissue specimen.

### 2.2. The Optical Imaging System

The commercial microscope (Zeiss LSM 510 META, Jena, Germany), equipped with a mode-locked femtosecond Ti: sapphire laser (Mira 900-F; Santa Clara, Coherent, America), used in this work has been described elsewhere [13, 14]. The system has two imaging modes: channel mode and lambda mode. In this study, the channel mode was used to achieve high-contrast TPEF/SHG images, by setting one channel corresponded to the wavelength range from 430 to 716 nm for collection of TPEF signals (color-coded red), while another channel covered the wavelength range from 389 to 419 nm for collection of SHG signals (color-coded green). The lambda mode was used to carry out the spectral images by collecting emission signals between 377 nm and 716 nm at intervals of 10.7 nm, and then emission spectrum can be obtained by averaging the intensity from each pixel within spectral image. The exciting power was 5–10 mw and the excitation wavelength was 810 nm. The scan time for an image with 512 × 512 pixels was 1.57 s and the measured resolution was 0.29 *μ*m per pixel.

## 3. Results and Discussion

### 3.1. Nonlinear Spectral Analysis of Normal Breast Tissue

**Figure 1:**
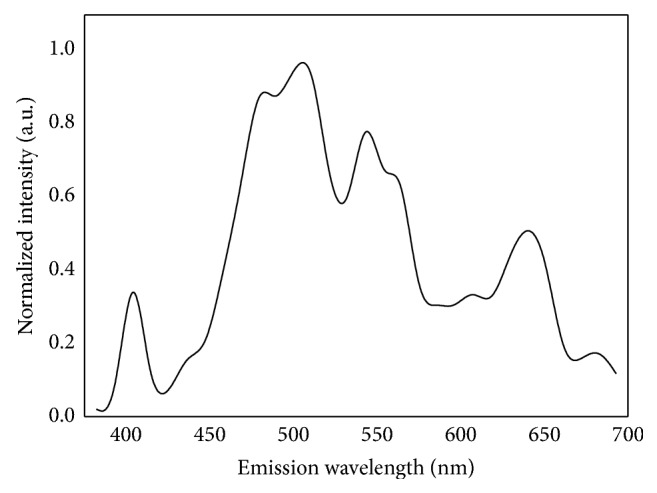
Normalized multiphoton emission spectrum of normal breast tissue, obtained with an excitation wavelength of 810 nm.

Firstly, the focus is on analyzing the origins of multiphoton signals in normal mammary gland and then determining whether the microstructures of breast tissue can be distinguished by MPM. Therefore, the emission spectrum of normal breast tissue was performed. The fresh tissue sections were excited at 810 nm excitation wavelength and the emission signals were collected by the lambda mode setting. The normalized multiphoton emission spectrum after subtraction of background was displayed in Figure 1. In general, there are six main peaks at 405, 470, 510, 540, 630, and 690 nm. According to previous publications [15–17], the fluorescence peaks at 470 and 540 nm are responsible for NADH and FAD, respectively, and the 405 nm peak (half of the 810 nm excitation wavelength) originates from collagen. Furthermore, the strong fluorescence peak at 510 nm is possible corresponding to cellular structural protein, which depends on the excitation/emission conditions used. Additionally, the fluorescence peak around 630 and 690 nm is attributed to porphyrin derivatives.

The origins of intrinsic signals in breast tissue were summarized in Table 1. According to the spectroscopic research, we can draw a conclusion that MPM can be used for identification of breast tissue without the use of exogenous contrast agents as there are many intrinsic signal sources.

### 3.2. MPM Identification of Normal Breast Tissue

**Figure 2:**
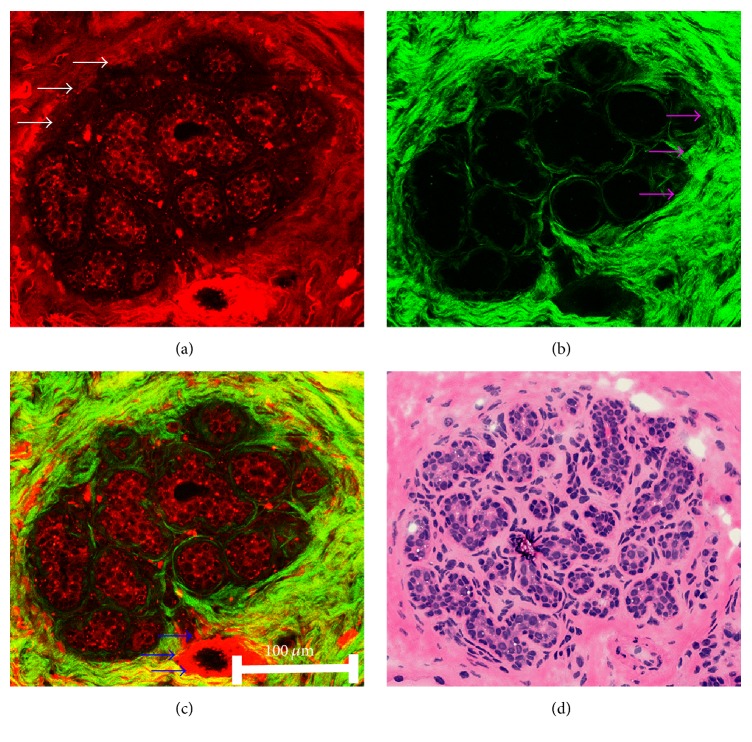
Representative TPEF/SHG images and corresponding H&E stained image of normal breast tissue. (a) TPEF image (color-coded red) of epithelial cells in lobule, collagen bundles, and blood vessel in stroma; (b) SHG image (color-coded green) of collagen bundles in stroma and collagen fibers inside lobule; (c) the overlaid image of TPEF and SHG; (d) the corresponding H&E stained image; white arrows, pink arrows, and blue arrows indicate mammary lobule, stroma, and the blood vessel, respectively.

Normal breast tissue consists of lobules and stroma. The mammary lobule (white arrows), which comprises groups of acini separated by collagen fiber, is visualized distinctively via their strong TPEF signals (Figure 2(a)). Epithelial cells inside acinus are distinctly observed based on the fluorescent cytoplasm and nonfluorescent nuclei. The stroma (pink arrows) mostly composed of collagen bundles is displayed well by its comparable TPEF and SHG signals (Figure 2(b)). Remarkably, the blood vessel (blue arrows) is also clearly identified in stroma based on strong TPEF signals in the overlaid TPEF/SHG image (Figure 2(c)). The details of lobular and stromal architecture readily correlate with H&E stained image (Figure 2(d)).

### 3.3. MPM Identification of Fibroadenoma of Breast

**Figure 3:**
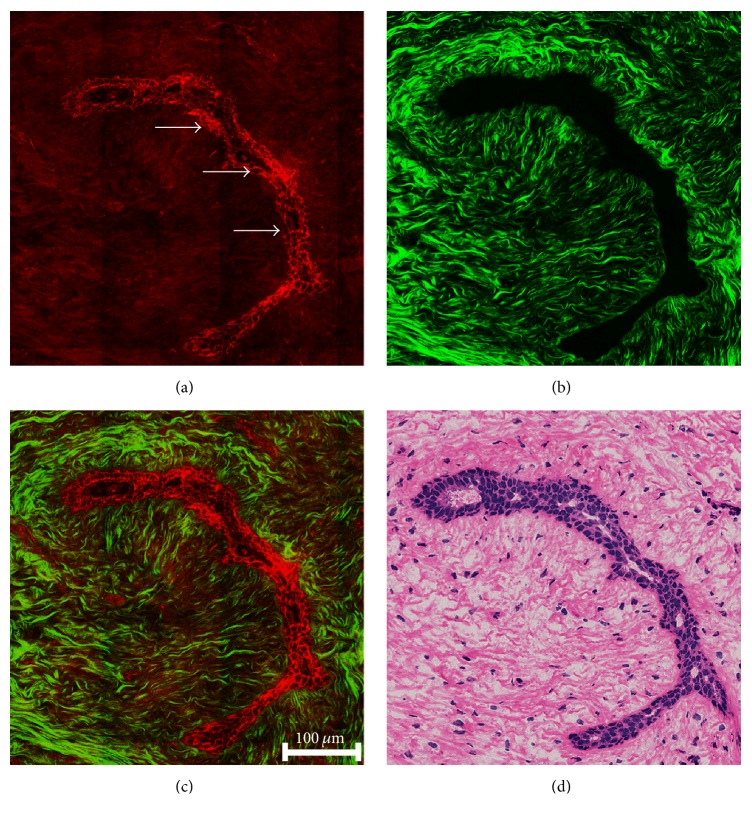
Representative TPEF/SHG images and corresponding H&E stained image of fibroadenoma of breast. (a) TPEF image (color-coded red) of compressed duct (white arrows); (b) SHG image (color-coded green) of collagen fibers surrounding duct; (c) the overlaid image of TPEF and SHG; (d) the corresponding H&E stained image.

Figure 3 shows the representative TPEF/SHG image and the corresponding H&E stained image of fibroadenoma of breast. The compressed mammary duct (white arrows) generates strong TPEF signals because of epithelial cells lining the outline of duct (Figure 3(a)). The collagen fiber around duct emits strong SHG signals due to their noncentrosymmetric molecular structure (Figure 3(b)). SHG image clearly reveals the significant alterations in the morphology and distribution of collagen fiber. Compared to normal breast tissue, collagen fiber increases in density, appears less organized, and becomes shorter. The interaction between compressed duct and surrounding collagen fiber is showed in Figure 3(c), which facilitates the pathological assessment of fibroadenoma. The details of cellular and stromal architecture readily correlate with H&E stained image (Figure 3(d)).

### 3.4. MPM Identification of Fibrocystic Diseases of Breast

**Figure 4:**
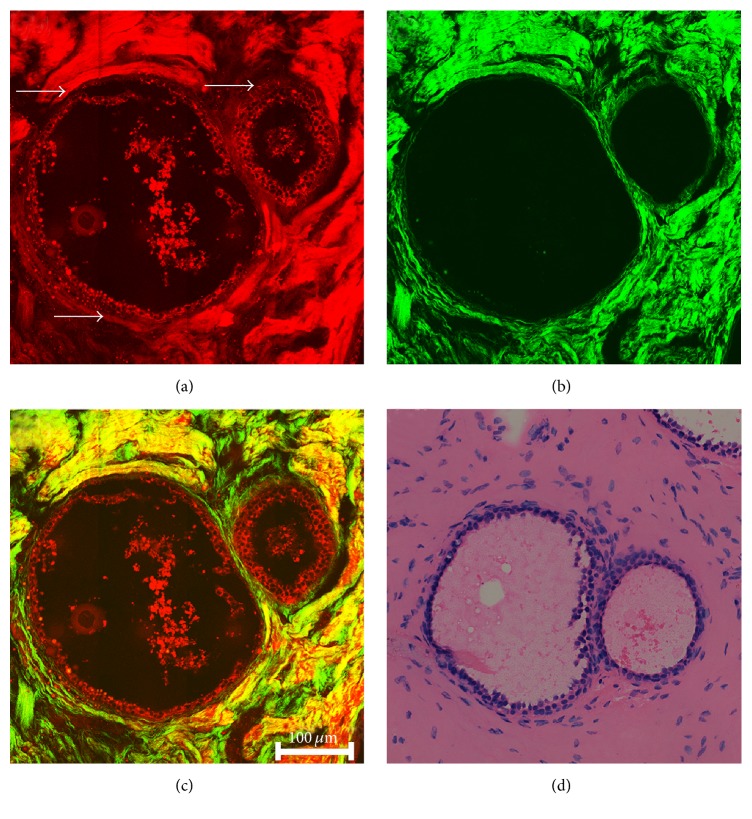
Representative TPEF/SHG images and corresponding H&E stained image of fibrocystic diseases of breast. (a) TPEF image (color-coded red) of the cysts filled with cystic fluid (white arrows); (b) SHG image (color-coded green) of collagen fibers surrounding cysts; (c) the overlaid image of TPEF and SHG; (d) the corresponding H&E stained image.

Figure 4 exhibits the representative TPEF/SHG image and the corresponding H&E stained image of fibrocystic diseases of breast. The cysts (white arrows) are lined by a small number of epithelial cells, with an enlarged lumen (Figure 4(a)). The lumen is filled with cystic fluid rather than cancerous or benign lump of cells, which can be distinctly observed based on strong TPEF signals. The collagen fibrous tissues surrounding the cysts are denser compared to normal breast tissue, which has comparable TPEF and SHG signals (Figure 4(b)). The architecture of cysts can be exhibited from the overlaid TPEF/SHG image (Figure 4(c)), which also readily correlate with H&E stained image (Figure 4(d)).

MPM diagnostic features can be clearly extracted by comparing the MPM images from normal breast tissue, fibroadenoma of breast, and fibrocystic disease of breast. As illustrated by Table 2, MPM has the ability to reveal morphological changes in both cellular feature and extracellular matrix (ECM) architecture, by combining TPEF signals from epithelial cells and SHG signals from collagen-rich ECM. It demonstrates that MPM is an advanced optical technology capable of generating information comparable to histopathology, allowing for diagnosing breast benign diseases as well as breast cancer at the molecular lever.

Breast is more superficial and particularly accessible to MPM technique. Along with MPM probes, GRIN lenses, and photonic crystal fibers [18–22], MPM can be integrated into the intra-fiberoptic ductoscopy or transdermal biopsy needle which will allow providing in vivo diagnosis of breast diseases, hence avoiding repeated needle or surgical biopsy, reducing patient anxiety in awaiting results, and expediting the scheduling of procedures in cases of carcinoma. Once MPM serves as advanced breast imaging technology for clinical applications like X-ray mammography, breast ultrasonography, MRI, and fiberoptic ductoscopy, diagnosing breast benign diseases will probably become its main application as well as breast cancer. This study provides the groundwork for the further use of MPM to perform such real-time noninvasive diagnosis of breast diseases.

## 4. Conclusion

In this study, MPM was used to image normal breast tissue, fibroadenoma lesion, and fibrocystic disease. Experimental results demonstrate that MPM has the capability to provide structural information comparable to histopathology, allowing for identifying the microstructure of lobule and stroma in normal breast tissue, the interaction of compressed ducts with surrounding collagen fiber in fibroadenoma, and the architecture of cyst filled with cystic fluid in fibrocystic disease, based on endogenous TPEF and SHG signals. With integration of MPM into clinical imaging system, it has the potential to make a real-time histological diagnosis of breast diseases in vivo.

## Figures and Tables

**Table 1:** The origins of intrinsic signals in breast tissue.

TPEF signals	SHG signals
(1) NAD(P)H and flavins, in epithelial myoepithelial cells in ducts	(1) Collagen fibers in basement membrane
(2) Collagen bundles in extracellular matrix	(2) Collagen bundles in extracellular matrix
(3) Collagen, elastin, in vessel stroma	(3) Collagen in vessel wall

**Table 2:** MPM diagnostic features of normal breast tissue, fibroadenoma lesion, and fibrocystic disease.

Breast tissues	MPM diagnostic features
Normal breast tissue	(1) Epithelial cells inside acinus generate strong TPEF signal, showing the regular arrangement and uniform cell size (2) Collagen fibers inside lobule emit strong SHG signal, exhibiting circular morphological feature

Fibroadenoma lesion	(1) Mammary ducts are distinctly compressed (2) Collagen fiber increases in density relatively, appears less organized, and becomes shorter

Fibrocystic disease	(1) The cysts are visualized with lumen filled with cystic fluid (2) The collagen fibrous tissues surrounding the cysts are denser compared to normal breast tissue
